# Polymorphisms and features of cytomegalovirus UL144 and UL146 in congenitally infected neonates with hepatic involvement

**DOI:** 10.1371/journal.pone.0171959

**Published:** 2017-02-21

**Authors:** Gangqiang Guo, Liang Zhang, Sisi Ye, Yingying Hu, Baoqing Li, Xiangwei Sun, Chenchen Mao, Jianfeng Xu, Yiping Chen, Lifang Zhang, Xiangyang Xue

**Affiliations:** 1 Department of Microbiology and Immunology, Institute of Molecular Virology and Immunology, Institute of Tropical Medicine, Wenzhou Medical University, Wenzhou, China; 2 Department of General Surgery, First Affiliated Hospital, Wenzhou Medical University, Wenzhou, China; 3 Department of Obstetrics and Gynecology, Second Affiliated Hospital, Wenzhou Medical University, Wenzhou, China; 4 Department of Laboratory Medicine, Second Affiliated Hospital & Yuying Children's Hospital, Wenzhou Medical University, Wenzhou, China; 5 Department of Pediatric Infectious Diseases, Second Affiliated Hospital & Yuying Children's Hospital, Wenzhou Medical University, Wenzhou, China; University of San Francisco, UNITED STATES

## Abstract

Human cytomegalovirus is a significant agent of hepatic involvement in neonates. In this study, we investigated the polymorphisms and features of the viral genes UL144 and UL146 as well as their significance to congenital hepatic involvement. In 79 neonates with congenital cytomegalovirus infection and hepatic involvement, full length UL144 and UL146 were successfully amplified in 73.42% and 60.76% of cases, respectively. Sequencing indicated that both genes were hypervariable. Notably, UL144 genotype B was highly associated with aspartate aminotransferase (*P* = 0.028) and lactate dehydrogenase (*P* = 0.046). Similarly, UL146 genotype G1 and G13 were significantly associated with CMV IgM (*P* = 0.026), CMV IgG (*P* = 0.034), alanine aminotransferase (*P* = 0.019), and aspartate aminotransferase (*P* = 0.032). In conclusion, dominant UL144 (genotype B) and UL146 (genotype G1 and G13) genotypes are associated with elevated levels of enzymes and CMV IgM and IgG of cytomegalovirus infection.

## Introduction

Human cytomegalovirus belongs to the subfamily *Betaherpesvirinae* in family *Herpesviridae*, and is congenitally transmitted to about 1. 8% of neonates in China [1], and to 0. 2−2.2% of newborns in other countries [2]. About 11% of congenitally infected infants born alive are symptomatic and present multisystemic or fatal disease [1], including hepatosplenomegaly, petechiae, megacolon, microcephaly, neurodevelopmental disorders, and hepatic involvement. In particular, the virus tends to infect the reticuloendothelial system, especially the liver [3]. Although 85−90% of infected neonates do not show clinical evidence of infection, they may develop several clinical outcomes in following years, including motor deficits, ocular abnormalities, and hearing loss [4]. Why only some neonates infected with HCMV, but not all population, develop symptoms is unknown [5]. Although the host immune system is believed to largely determine the outcome of infection, sequence polymorphisms in the infecting strains are also thought to be associated with outcome and tissue tropism [6, 7].

Human cytomegalovirus is one of the largest human viruses. It carries approximately 230−235 kb of double-stranded DNA and >200 predicted open reading frames [8–10]. Notably, the laboratory strain AD169 lacks the UL/b' sequence, in contrast to the low-passage clinical strain Town and several other low-passage clinical isolates. This fragment contains at least 19 open reading frames, including UL133−UL151, and is dispensable for growth *in vitro*, but may be essential for viral infectivity and pathogenicity *in vivo* [11]. Of these, UL144 is expressed early in lytic infection, and encodes a structural homologue of a herpesvirus receptor that mediates viral entry. Poole *et al*. found that, although IE86 represses the UL144-mediated activation of a synthetic NF-B promoter, it is unable to block UL144-mediated activation of the CCL22 promoter, and this lack of responsiveness to IE86 appears to be regulated by binding of the CREB transcription factor [12]. Moreover, Cheung *et al*. found that UL144 binds Ig superfamily member B and T lymphocyte attenuator (BTLA), but not LIGHT, and inhibits T cell proliferation, selectively mimicking the inhibitory co-signaling function of herpesvirus entry mediator (HVEM) [13]. Four UL144 transcripts have been identified in infected cells as variously regulated 3'-coterminal transcripts of 1,300, 1,600, 1, 700, and 3,500 nucleotides, while the largest transcript initiated from within the UL141 open reading frame includes UL141-UL145 [14]. On the other hand, UL146 encodes a viral α (CXC)-chemokines (vCXCL-1), a sufficiently functional chemokine, that elicits chemotaxis and mobilizes calcium [15, 16]. In infected endothelial cells, the viral chemokine recruits neutrophils via cellular CXCR1 and CXCR2 receptors, and the cells subsequently transport the virus to uninfected endothelial cells. In this manner, a large population of infected endothelial cells is maintained [17].

Remarkably, unrelated strains cluster into defined UL146 genotypes, of which 14−15 have been catalogued [18, 19]. Accordingly, UL146 diversity impacts binding affinity, receptor targeting, activation of peripheral blood neutrophils, and, hence, virus dissemination and pathogenesis [20]. Indeed, UL144 and UL146 are some of the most polymorphic genes in many clinical isolates [7, 15, 18, 21–24]. The relationship between these polymorphisms and several symptoms have been evaluated in various studies with inconclusive or contradictory results [25]. However, it has not been reported in congenitally infected neonates with hepatic involvement. Therefore, we investigated UL144 and UL146 polymorphisms and genotypes in 79 newborn infants with congenital cytomegalovirus infection and hepatic involvement, and explored their correlation with clinical outcome.

## Materials and methods

### Study population and sample collection

From November 2014 to May 2016, 79 newborn infants with congenital cytomegalovirus infection and hepatic involvement were recruited at Second Affiliated Hospital of Wenzhou Medical University, Wenzhou, China. Average age was 6 days, with a median of 1.8 days and a range of 1–13 days.

Congenital HCMV infection is diagnosed when a newborn is confirmed to be infected by HCMV within 14 days (including the 14^th^ day) after birth based on one of the following standards used to define HCMV infection: the virus copies of the blood or urine of patients detected by fluorogenic quantitative PCR is >500copies/mL; serological test demonstrates CMV IgG >1.00 U/mL or CMV IgM >1.00 COI.

Cytomegalovirus hepatitis was diagnosed according to criteria set at the National Infant Virus Hepatitis Prevention and Cure Symposium in China. The criteria consist of (i) several significant indicators of hepatic involvement, (ii) detection of cytomegalovirus DNA in urine or blood within 2−3 weeks after birth, (iii) absence of Epstein-Barr virus and hepatitis A, B, C, D, and E, and (iv) exclusion of metabolic disorders, alcoholic hepatitis, drug-induced hepatitis, familial cholestasis, autoimmune hepatitis, or idiopathic neonatal hepatitis. Indicators of hepatic involvement include alanine aminotransferase (ALT) > 40 units/L, aspartate aminotransferase (AST) > 35 units/L, and lactate dehydrogenase (LDH) > 245 units/L. Presence of cytomegalovirus DNA was tested by fluorescence quantitative PCR (Applied Biosystems 7500 Real−Time PCR System, Foster City, CA, USA).

Sixty neonates tested positive for serum CMV IgG against cytomegalovirus, while 25 tested positive for CMV IgG and IgM. CMV IgG and IgM were detected by chemiluminescence immunoassay, following the manufacturer’s instructions (Roche Cobas 8000 Analysis System and Auxiliary Kit, Basel, Switzerland). Sediments from 5 mL urine were collected and stored at −80°C until use. Urine samples were collected during standard examination at admission, and written informed consent was obtained from the parents of the newborn infants who participated in the study. The corresponding author, Xiangyang Xue, was responsible for anonymizing the data collected from participants and this anonymization procedure was approved by the Ethics Committee that approved the study. All data for this study were used and analyzed in strictly anonymous form, according to the code of conduct for medical research approved by the hospital’s Medical Ethical Committee. The Medical Ethical Committee of the hospital of the Second Affiliated Hospital of Wenzhou Medical University approved the consent procedure and current study. Baoqing Li and Yiping Chen, two of the authors of this study, involved in collection of the participant data.

### DNA extraction, polymerase chain reaction, and sequencing

Viral DNA was isolated from urine sediments using TIANamp Genomic DNA Kit (Tiangen, Beijing, China) according to the manufacturer’s protocol and was eluted in 100 μL elution buffer. DNA concentration and purity were assessed by spectrophotometry (Beckman, Fullerton, CA, USA), and samples were stored at -20°C until use. UL144 was detected using primers designed to generate a 740bp product [19] after denaturation at 95°C for 2 min, 40 cycles at 95°C for 30 s, 55°C for 30 s, 72°C for 1 min, and extension at 72°C for 10 min [19]. UL146 was detected using primers designed by Hassan-Walker *et al*. [15]. These primers amplified a 721bp fragment encompassing UL146 and flanking sequences from UL145 and UL147 in reactions consisting of denaturation at 95°C for 10 min, 40 cycles at 94°C for 30 s, 55°C for 30 s, 72°C for 30 s, and final extension at 72°C for 10 min [15]. Primer sequences are listed in Table 1. PCR products were cloned into pEASY-T1 Cloning Vector (TransBionovo, Beijing, China) using pEASY-T1 Cloning Kit (TransBionovo, Beijing, China), and transformed into Trans-T1 Phage-Resistant Chemically Competent Cells (TransBionovo, Beijing, China) according to the manufacturer's instructions. At least ten colonies were sequenced in both directions on a 3730xl DNA Analyzer (Applied Biosystems), using the universal primers M13 and T7. Clones were analyzed in Chromas and verified by Basic Local Alignment Search Tool [23].

**Table 1:** Primers and reaction conditions for amplifying full length viral ORFs.

	Sequences		PCR conditions			
	Forward primer(5'-3')	Reverse primer(5'-3')	Annealing temperature(°C)	Time(s)	Cycles	Produc(bp)
UL144	CGTATTACAAACCGCGGAGAGGAT	CTCAGACACGGTTCCGTAAAGTG	55	30	40	740
UL146	CCGGGAATACCGGATATTACG	CAGCACTTCCTGACGATTGC	55	30	40	721

### Template DNA used for the sensitivity tests of UL144 and UL146 PCR

HCMV clinical strains separated in our laboratory were used as templates with full length primers of UL144 and UL146 (Table 1) to amplify target genes. The amplified products were electrophoresed on a 1.5% agarose gel containing ethidium bromide (EB) and the bands were photographed. They were also sequenced to ensure that the sequence was correct. The correct PCR product was cloned into T vector. The concentration was determined and then converted to copy number. The vector was serially diluted to 10^4^, 10^3^, 10^2^, 10^1^, and 10^0^ copies. Serially diluted samples were used as templates to detect the sensitivity of the primers for the detection of UL144 and UL146; the emergence of target bands was used as a positive criterion.

### Sequence analysis

Nucleotide sequences were aligned and edited in BioEdit, and translated in Vector NTI. BioEdit was also used to analyze homology, which was scored based on identical and conserved amino acids [15]. Expasy, NetNGlyc 1.0 and interpro (EBI) were used to analyze protein physical and chemical properties. Sequence logos were generated with WebLogo (http://weblogo.berkeley.edu/examples.html). Clinical isolates were compared to reference sequences for UL144 and UL146 genotypes (GenBank: AF498086–AF498090), and to published sequences from Arav-Boger *et al*. [26], Waters *et al*. [27], Heo *et al*. [19], Mao *et al*. [5], Paradowska *et al*. [6], Arav-Boger *et al*. [28], and Stanton *et al*. [29]. UL144 was genotyped according to Arav-Boger *et al*. [30], while UL146 was genotyped according to the 14 genotypes described by Dolan *et al*. [8]. Neighbor-joining phylogenetic trees were generated in MEGA5.0 with 100 bootstrap trials. Bootstrap values ≥50% indicated significant clustering.

### Statistical analysis

Non-parametric Mann-Whitney U test was performed to compare clinical indicators in mixed infection and single virus strain patients. R × C chi-square test was performed to compare the distribution of UL144 and UL146 genotypes with published data. Nonparametric Kruskal-Wallis test was used to compare isoelectric points and molecular weights of UL144 and UL146. Potential linkage disequilibrium between genotypes was compared using Cohen's Kappa coefficient. The nonparametric Kruskal-Wallis test was performed to compare clinical indicators in neonates infected with various genotypes. For clinical indicators showing distribution differences, we further adopted Nemenyi test to compare the differences between each other. P values *<* 0.05 were considered significant. Data were analyzed in SPSS 16.0 (SPSS, Chicago, IL, USA).

## Results

### Frequency of UL144 and UL146 detection in neonates with congenital cytomegalovirus hepatic involvement

**Fig 1:**
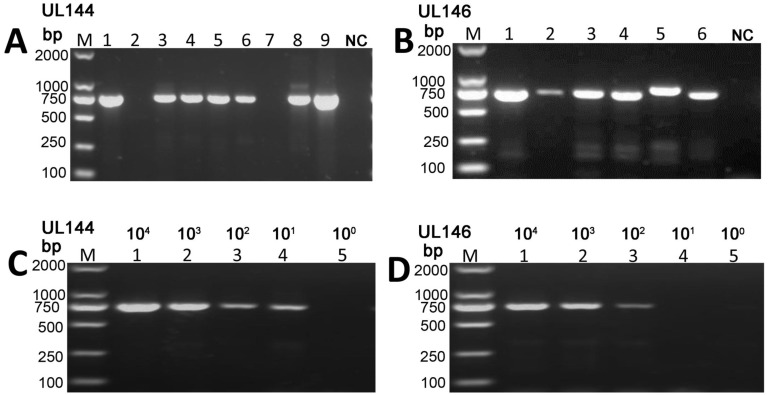
UL144 and UL146 genes in patients with congenital cytomegalovirus hepatic involvement. (A, B) PCR detection and identification of human cytomegalovirus (HCMV) UL144 and UL146 in urinary sediment, with expected size of 740 bp and 721 bp, respectively. M, 100 bp DNA marker; (A) Lane 1–9, UL144, from the sample 2H, 5H, 6H, 10H, 12H, 14H, 22H, 23H, and 25H, respectively; Lane 10, negative control (NC, HCMV negative urine sample); (B) Lane 1–6, UL146, from the sample 58H, 59H, 60H, 61H, 62H, and 63H, respectively; Lane 7, negative control (NC, HCMV negative urine sample). (C, D) PCR Sensitivity. UL144 and UL146 were amplified by PCR, and separated on 1.5% agarose. Lane 1–5, reactions with 10^4^, 10^3^, 10^2^, 10^1^, and 10^0^ copies, respectively; M, D2000 DNA marker.

As shown in Fig 1A and 1B, sharp bands were generated with each primer pair from samples infected with cytomegalovirus, but not from negative controls (HCMV negative urine sample). Sequencing confirmed the specificity of PCR reactions (S1 Fig), whose sensitivity was determined to be 10^1^ copies/reaction for UL144 and 10^2^ copies/reaction for UL146 (Fig 1C and 1D). Bioinformatic analysis of 250 strains indicated that homology was 87.5−100% and 91.3−100% in the forward and reverse primer regions of UL144, respectively. In particular, the last six 3' nucleotides of the UL144 primer were completely conserved, except in four strains, to which the primer did not match well. Therefore, the UL144 primer can theoretically amplify 98.4% (246/250) of the strains analyzed. A similar analysis of 265 strains indicated that the homology in the UL146 forward and reverse primer regions was 90.4−100% and 100%, respectively, with the last six nucleotides of the primer being completely conserved except in two strains. Hence, the UL146 primer should theoretically amplify 99.25% (263/265) of the strains.

Out of the 79 samples, UL144 and UL146 were successfully amplified and sequenced by PCR in 58 and 48 of patients, respectively (Table 2). The genes were not amplified in one-third of the samples. For specimens without amplification, we tested redesigned UL144 and UL146 primers or primers reported by others [7, 31] which also failed, probably due to low DNA copy number or poor PCR sensitivity.

**Table 2:** The frequency of UL144 and UL146 and mixed infections in neonates with congenital cytomegalovirus hepatic involvement.

	Positive rate	A single virus infection	Mixed infection
UL144	58/79(73.42%)	49/58(84.48%)	9/58(15.52%)
UL146	48/79(60.76%)	42/48(87.5%)	6/48(12.5%)

### Features and genotypes of UL144 in clinical isolates

**Fig 2:**
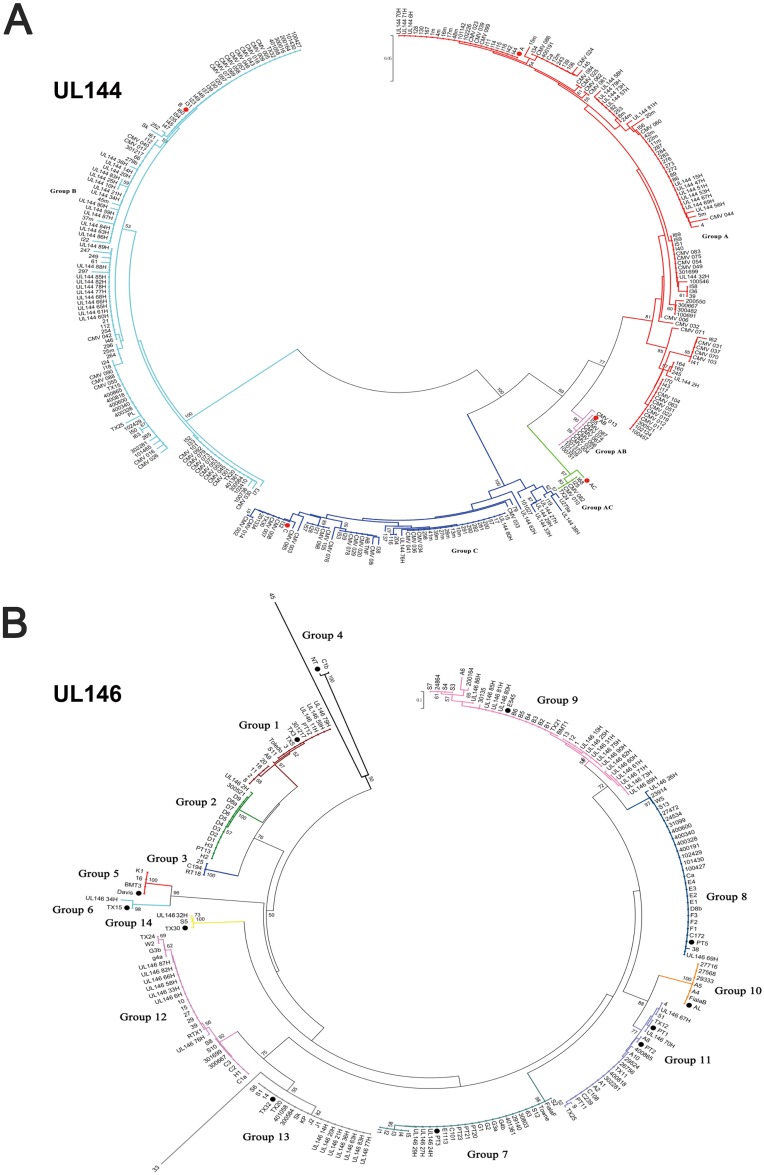
Phylogenetic analysis of the UL144 and UL146 amino acid sequence. Phylogenetic analysis of UL144 (A) and UL146 (B) based on amino acid sequences aligned with Clustalx. Representative sequences from genotypes A, B, and C (GenBank accession numbers AF498086—AF498088) were included as references for UL144 gene (the red dot). G1 (PT12), G2 (PT13), G6 (TX15), G7 (PT20), G8 (PT5), G9 (E545), G11 (PT1), G12 (TX24), G13 (TX32), and G14 (TX30) were included as references for UL146 gene (the black dot). Fig 2A and 2B contains not only previously reported sequences (254 for Fig 2A and 163 for Fig 2B and their study, which was described in Section Materials and methods), but also this study population. Our sequences were named “UL144 XXH” or “UL146 XXH”(XX, stood for numbers) in Fig 2A or 2B. Pairwise evolutionary distances were estimated using Poisson model, and trees were constructed by a neighbor-joining method implemented in MEGA5.0. The reliability of each tree topology was estimated from 100 bootstrap replicates.

UL144 genes were generally classified into 5 genotypes,named A,B,C,AB,and AC. Two hundred fifty four previously reported UL144 sequences [5, 19, 26, 27] were retrieved and analyzed in the MEGA 5.0 software. As shown in Fig 2A, we found 97 in group A, 92 in group B, 49 in group C, 11 in group AB, 5 in group AC. Comparatively, according to our UL144 sequencing results, 49 out of 58 patients were classified as A, B, and C, among which genotype B accounted for approximately half of the patients (51.02%), while genotypes AB and AC were not detected (S1 Table). Our sequences were named “UL144 XXH” (XX, stood for numbers) in Fig 2A. In addition, the remaining 9 patients were defined as mixed infection, which are described in the “features and genotypes of mixed infections” section below. We previously reported the distribution of UL144 genotypes in congenital infection asymptomatic patients, which showed that the genotypes A and B were detected, but genotypes C, AB, and AC were not [32].

**Fig 3:**
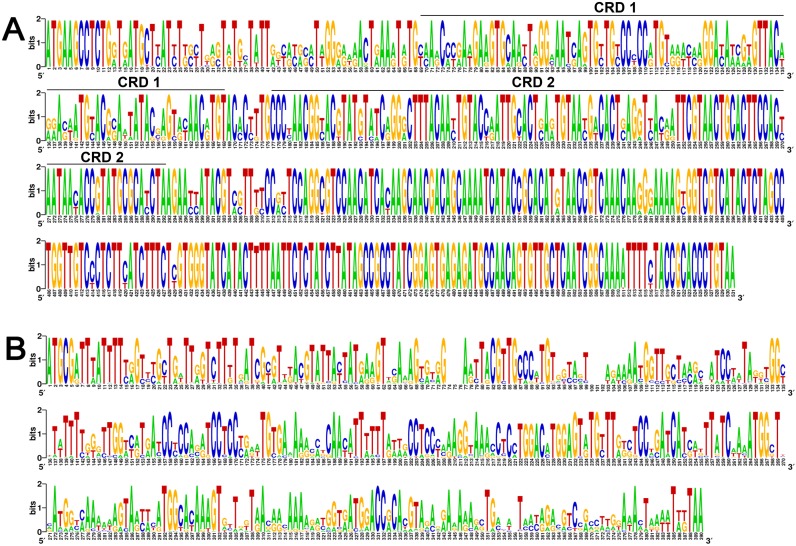
DNA logos of UL144 and UL146. A logo represents each column of the alignment as a stack of letters, the height of each letter being proportional to the observed frequency of the nucleotide in 49 UL144 (A) and 42 UL146 (B) of single virus strain. In addition, the overall height of each stack is proportional to sequence conservation at that position, as measured in bits. The letters of each stack are ordered from most to least frequent, so that the consensus sequence can be inferred from the tops of the stacks. CRD, Cysteine-rich domains.

UL144 gene polymorphism of the Towne strain and of 49 sequences was then observed, with homology ranging from 80% to 100% at the nucleotide level, and from 77.7% to 100% at the amino acid level (S2 Table). Logos of UL144 gene sequence alignment showed the variation was concentrated on the 5' half of the gene, especially the CRD 1 domain. (Fig 3A).

**Fig 4:**
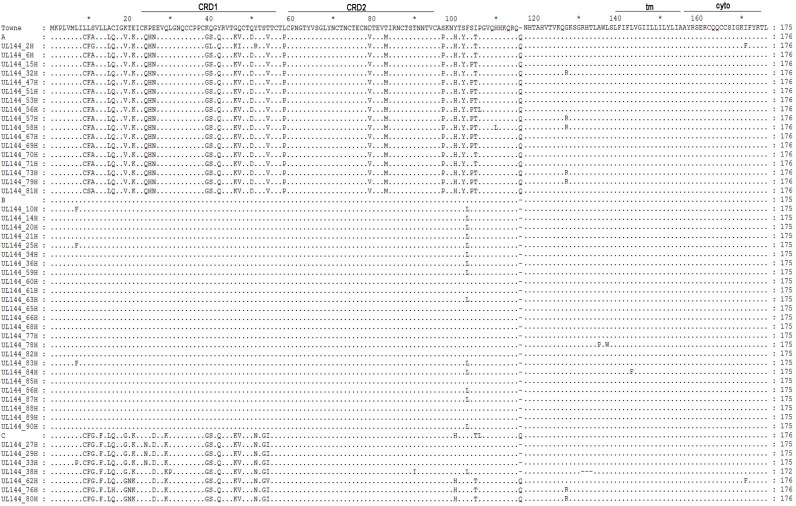
Alignment of amino acid sequences and comparison of post-translational modification motifs in UL144. UL144 amino acid sequences, along with representative sequences of genotypes A, B, and C (GenBank accession numbers AF498086-AF498088) as reference. Cysteine-rich domains (CRD), transmembrane region (tm), and cytoplasmic tail (cyto) are marked.

Amino acid sequence polymorphism of the same type was also analyzed. Comparing with the Towne strain, we found significant differences in genotypes A (79.5−80.6% identity) and C (78.8−82.2% identity) (Fig 4A). Moreover, genotype A isolates differed from the Towne strain by 25−27 amino acid substitutions, as well as by insertion of glutamine (Gln, Q) at position 116, so that the UL144 protein was 176 amino acids in length. Similarly, genotype C isolates differed from the Towne strain by 19−23 amino acid substitutions and by insertion of glutamine at position 116, as observed in 3/7 (42.86%) isolates. Furthermore, amino acids 131−133, corresponding to an arginine (Arg, R), a histidine (His, H), and a threonine (Thr, T), were deleted from genotype C isolates, so that predicted peptide sequences ranged from 172 to 176 amino acids. Missense mutations were the most common polymorphisms in both genotypes A and C. On the other hand, genotype B was strongly conserved, with amino acid homology as high as 97.7−98.8%. In addition to leucine (Leu, L) being mutated into phenylalanine (Phe, F) at position 7 or phenylalanine (Phe, F) being mutated into leucine (Leu, L), the mutation type was almost synonymous mutation, and the mutation rate was 56% (14/25) (Fig 4).

To evaluate whether the variation in amino acid sequence would influence the physical and chemical property of proteins, protein isoelectric points (IP) and molecular weights (MM) were predicted in the EXPASY database. The MW median was 19.63, 19.49, and 19.30 kDa, for genotype A, B, and C, and IP was 8.97, 8.87, and 9.04, respectively (S2A and S2B Fig) with obvious differences (P = 0.000).

Depending on the hypervariable amino acid sequences, we then analyzed the functional motifs of UL144 proteins. Most essential motifs such as *N*-glycosylation sites, *N*-myristoylation sites, protein kinase C (PKC) phosphorylation sites, and TNFR/NGFR cysteine-rich regions were present in all genotypes, although genotype C isolates contain two additional *N*-myristoylation sites due to mutation of valine 19 and serine 53 to glycine (Table 3). Notably, only genotype B strains contained bacterial Ig-like domain 1, a prokaryotic site for binding membrane lipoprotein lipids (Prokar_Lipoprotein), and a CTCHY zinc finger (Table 3). Furthermore, even though the UL144 genotype in congenitally infected neonates with hepatic involvement possessed the functional site of the genotype in congenital infection asymptomatic neonates, the UL144 type A and type B in the former had one more PKC phosphorylation site (49^th^–51^th^ amino acids) and Bacterial Ig-like domain 1 (1^th^–8^th^ amino acids), respectively [32]. In any case, the UL144 protein from all genotypes consists of two cysteine-rich domains (CRD1 and CRD2), one transmembrane domain (tm), and one cytoplasmic domain (cyto), as illustrated in Fig 4. In comparison to the Towne strain, CRD1 was more variable in genotypes A and C, but was significantly more conserved in genotype B. However, the tm and cyto were strongly conserved in all genotypes (Fig 4).

**Table 3:** Functional motifs in the HCMV UL144 protein from different UL144 genotypes.

Functional Site	Position of amino acid		
	Type A	Type B	Type C
*N*-glycosylation sites	61–64,70–73,78–81,86–89,91–94,99–102,117–120	61–64,70–73,78–81,86–89,91–94,99–102,116–119	61–64,70–73,78–81,86–89,91–94,99–102,116–119
*N*-myristoylation	41–46,62–67	62–67	19–24,41–46,53–58,62–67
PKC phosphorylation	49–51,123–125,129–131,159–161	83–85,122–124,128–130,158–160	83–85,122–124,128–130,158–160
TNFR_NGFR_1	59–95	59–95	59–95
TNFR_NGFR_2	58–95,22–56	15–56,58–95	58–95
Cysteine-rich regions	34–77	34–77	34–77
NCD3G	55–76	55–76	55–76
TNFR_c6	59–95	59–95	59–95
Bacterial Ig-like domain 1	\	1–8	\
Prokar_Lipoprotein	\	1–16	\
CTCHY zinc finger	\	30–96	\

The number represents the location of amino acid. The first amino acid encoded by the initiation codon of UL144 gene was defined as 1 to confirm the amino acid location of each functional site. “/” represents homologous genotype of UL144 with no such functional site.

### Features and genotypes of UL146 in clinical isolates

**Fig 5:**
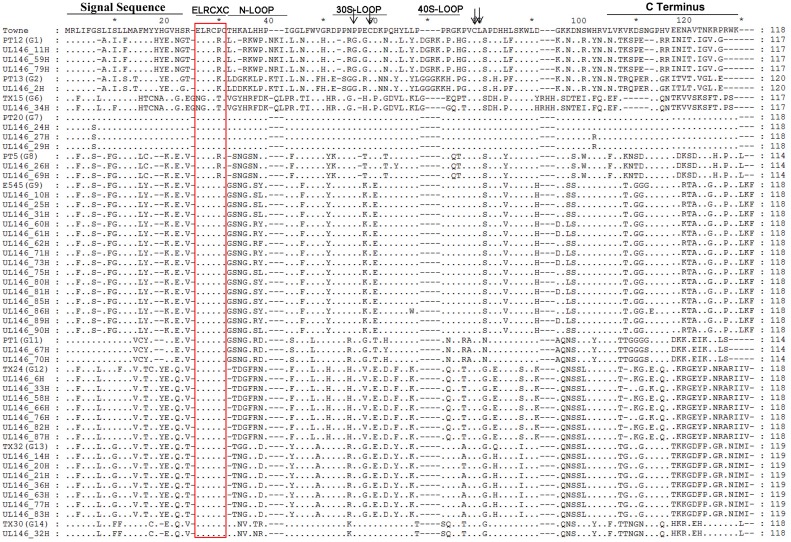
Alignment of amino acid sequences and comparison of post-translational modification motifs in UL146. UL146 amino acid sequences, along with representative sequences of genotypes G1 (PT12), G2 (PT13), G6 (TX15), G7 (PT20), G8 (PT5), G9 (E545), G11 (PT1), G12 (TX24), G13 (TX32), and G14 (TX30) as reference. The functional motifs ELRCXC (X represent the amino acid R or T), N-loop, 30s loops, 40s loops, and c terminus are marked at the top. A dot indicates identity, and a dash denotes deletion. Numbers above the sequences indicate amino acid position. Strains are listed according to sequence groups determined by phylogenetic analysis. Arrows above the sequences represent the amino acid position of 56, 59, 79, and 80, respectively. The red frame indicates the region of ELRCXC.

UL146 genes were generally classified into 14 genotypes, named G1−G14 [8]. Hundred sixty three previously reported UL146 sequences were retrieved [6, 19, 28, 29] and analyzed in the MEGA 5.0 software. As shown in Fig 2B, we found 13 in group 1, 13 in group 2, 3 in group 3, 3 in group 4, 4 in group 5, 1 in group 6, 23 in group 7, 25 in group 8, 19 in group 9, 7 in group 10, 20 in group 11, 18 in group 12, 12 in group 13, and 2 in group 14. Comparatively, we successfully sequenced and genotyped 42 out of 48 UL146 fragments into genotypes G1, G2, G6-G9, and G11−G14 (Fig 2B). G9 was the most prevalent, and was found in 15 patients. G12 and G13 were found in seven cases each, while G1 and G7 were detected in three cases each. G8 and G11 infected two newborns each, and G2, G6, and G14 were found in one patient each, while G3−G5 and G10, which were described previously [8], were not detected. Our sequences were named “UL146 XXH” (XX, stood for numbers) in Fig 2B. In addition, the remaining 6 out of 48 patients were defined as mixed infection, which are described in the “features and genotypes of mixed infections” section below. UL146 gene polymorphism of the Towne strain and total 42 sequences was then observed. Like UL144, UL146 was hypervariable, with homology among strains ranging from 40% to 100% at the nucleotide level, and from 18.5% to 100% at the amino acid level (S2 Table). Comparison with the Towne strain indicated that polymorphisms were distributed throughout the entire coding region except in G7 (Fig 5). Logos of UL146 gene sequence alignment also showed that the variation was distributed throughout the entire coding region (Fig 3B).

The predicted amino acid sequences were compared among 10 groups (Fig 5). The estimated size of the UL146 protein ranged from 114 to 120 amino acids. Moreover, the mature forms without the signal sequence and the reference sequences contain between 93–98 residues [19]. Missense mutations in the ELR motif, including glutamate (E) → asparagine (N) and leucine (L) → glycine (G) substitutions, were observed, and may have far-reaching effects, as the motif is essential for chemokine activity and receptor binding [19, 33, 34]. The ELRCXC motif also regulates angiogenesis [35]. The Towne strain contained an ELRCPC motif, as did all 35 strains of genotype G7, G9, and G11-G14, although amino acid homology among these 35 strains was 42.5–100%. On the other hand, a G2 strain contained an ELRCKC motif, while five G1 and G8 strains contained an ELRCRC motif. Notably, a G6 strain contained an NGRCTC motif without an ELR motif (Fig 5). The amino acid homology between G6 and other genotypes ranged from 18.5% to 30.4%, suggesting a clear difference in protein sequence.

In comparison to host chemokines, CXCL-1 and CXCL8, UL146 contained about 25 additional residues at the C terminus, as previously observed by Heo *et al* [20]. Except proline (P) 56, which was not conserved in cytomegalovirus isolates, the arginine (R) in the ELR motif, two cysteines (C) in the N terminus at positions 59 and 79, and a leucine (L) at position 80 were conserved in UL146 and other host chemokines (Fig 5) [17, 20]. Homology in the ELR motif, N-loop, and C terminus also implies differences in chemokine receptor binding and functional response [20].

Like UL144, to evaluate whether the variation in amino acid sequence would influence the physical and chemical property of UL146 proteins, molecular weights (MW) and protein isoelectric points (IP) were predicted in the EXPASY database. The predicted molecular weight of the UL146 protein was the lowest in G13 (10.73 kDa) and the highest in G1 (11.20 kDa), whereas the IP was the smallest in G12 (9.30) and the largest in G1 (9.89) (S2C and S2D Fig) with a significant difference (P = 0.000).

Depending on the hypervariable amino acid sequences, we then analyzed the functional motifs of UL146 proteins. G1 was predicted to contain an amidation site, three N-glycosylation sites, a tyrosine kinase phosphorylation site, and a protein kinase C phosphorylation site. G7 was predicted to contain an amidation site and two protein kinase C phosphorylation sites, while G9, G12, and G13 contained bacterial Ig-like domain 1. These results suggest that UL146 is post−translationally modified in different ways among the five major genotypes (Table 4).

**Table 4:** Functional motifs in the HCMV UL146 protein from different UL146 genotypes.

Functional Site	Position of amino acid				
	G1	G7	G9	G12	G13
Amidation	65–68	83–86	/	/	112–115
*N*-glycosylation sites	21–24,98–101,109–112	/	/	85–88	85–88
*N*-myristoylation	54–59	/	9–14	6–11,83–88	6–11,40–45,83–88
TYR kinase phosphorylation	88–95	/	/	/	/
PKC phosphorylation	/	31–33,111–113	/	95–97	95–97,105–107
Bacterial Ig-like domain 1	/	/	1–4	1–4	1–4

The numbers represent the location of amino acid. The first amino acid encoded by the initiation codon of UL146 gene was defined as 1 to confirm the amino acid location of each functional site. “/” represents homologous genotype of UL146 with no such functional site.

### Linkage of UL144 and UL146 sequence genotypes

Potential linkage disequilibrium between both the UL144 and UL146 genotypes was investigated in 37 single infected samples, both UL144 and UL146 genes were detected, from neonates with congenital cytomegalovirus hepatic involvement. The observation that 8 of 13 (61.54%) UL146 G9 were UL144 B's and that 7 of 7 (100%) UL146 G13 isolates were UL144 B's shows a linkage between UL144 and UL146 genotypes (Table 5). However, we did not observe a conformity between UL144 and UL146 variants (k = - 0.008, p = 0.739) in all patients examined.

**Table 5:** Analysis of linkage disequilibrium.

UL146 genotype	UL144 genotype			
	A	B	C	Total
G1	1	1	0	2
G2	1	0	0	1
G6	0	1	0	1
G7	0	0	2	2
G8	1	0	0	1
G9	3	8	2	13
G11	2	0	0	2
G12	2	3	2	7
G13	0	7	0	7
G14	1	0	0	1
Total	11	20	6	37

The numbers indicate the quantity of single infected patients (neonates with congenital cytomegalovirus hepatic involvement). No significant linkage between the UL144 and UL146 genotypes was found using Cohen's Kappa test (k = - 0.008, p = 0.739).

### Features and genotypes of mixed infections

**Fig 6:**
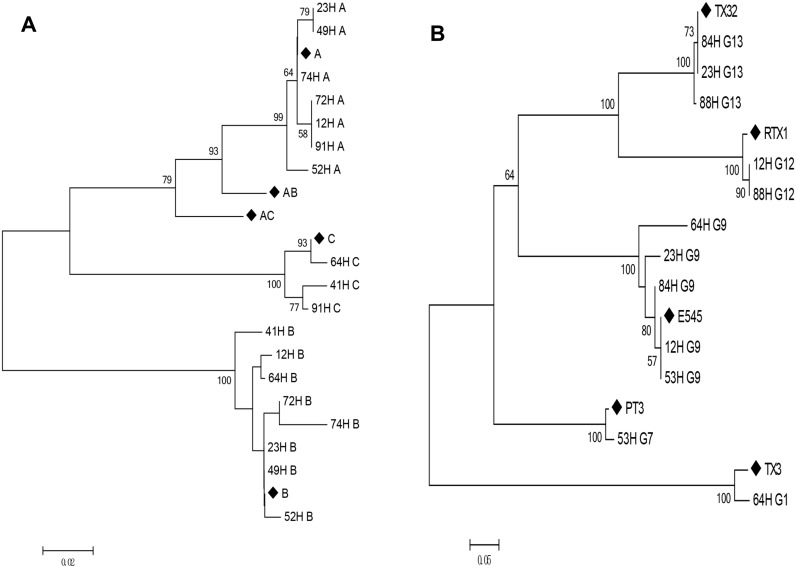
Phylogenetic analysis of the mixed infection amino acid sequences. A, Patients with multiple UL144 genotypes. B, Patients with multiple UL146 genotypes. Representative sequences (◆) as described in Fig 4.

**Fig 7:**
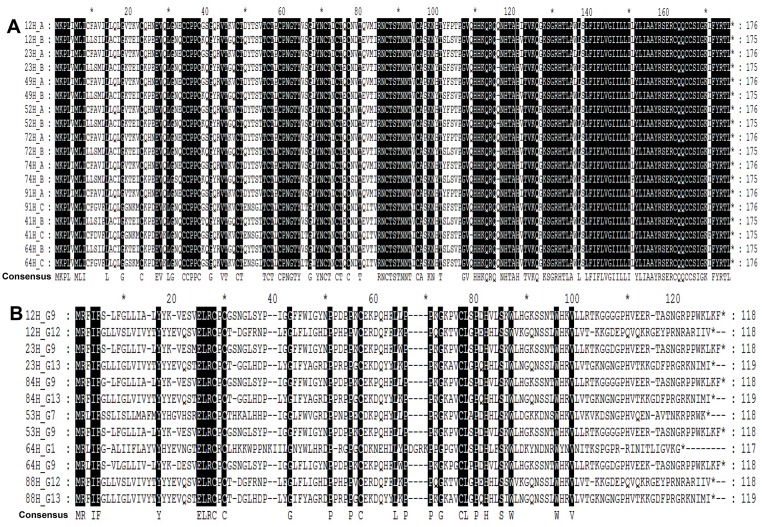
Alignment of the mixed infection amino acid sequences in UL144 and UL146 ORFs. A, amino acid sequences of multiple UL144 genotype in nine patients. B, amino acid sequences of multiple UL146 genotype in six patients. Consensus sequences are shown at the bottom. Numbers before “H_” means the patient code, while the letters after “H_” indicate the HCMV genotypes.

Two or more sequences were detected in the same samples more than once by sequencing after cloning, which indicated that these samples contained more than one HCMV strains. Moreover, alignment of amino acid sequence and clustering analysis also proved the presence of multiple UL144 or UL146 genotypes (S1 Table, Figs 6 and 7). Specimens from 9/58 (15.52%) of infants contained multiple UL144 genotypes, while 6/48 (12.5%) of specimens contained multiple UL146 genotypes (Table 2, S1 Table). Among 9 specimens with multiple UL144 genotypes, 6 samples presented mixed infection of type A and B, 2 samples presented mixed infection of type B and C, and 1 sample presented mixed infection of type A and C. Among 6 specimens with multiple UL146 genotypes, 2 samples presented mixed infection of type G9 and G13, one sample presented mixed infection of type of G9 and G12, one presented mixed infection of type of G7 and G9, one presented mixed infection of type of G1 and G9, and one presented mixed infection of type of G12 and G13. Three samples (12H, 23H, and 64H) showed mixed infection for UL144 and UL146 genes simultaneously. The results showed that the UL144 genotype of neonates with congenital cytomegalovirus hepatic involvement mixed infection was type A and B mainly. Moreover, 5/6 samples presented the G9 genotype for UL146 genotype of mixed infection. The feature of UL144 and UL146 gene polymorphism in mixed infective samples were the same as that in single infective samples.

Mixed infection, defined as the presence of multiple UL144 or UL146 genotypes, was observed in 12/79 (15.19%) neonates (S2 Table). We noted that most similar analyses in the past were based on virus isolates propagated in culture, a process that may have selected a single strain and, therefore, prevented detection of mixed infection. In contrast, we tested clinical specimens directly to prevent underestimation of virus diversity.

Moreover, correlation analysis of the mixed infection and single strain infection neonates for the clinical indicators was performed. The CMV IgM level in the group of newborns infected with a single virus strain displayed a median of 1.585 COI (interquartile range, 0.404 to 7.348) and statistically higher (P = 0.003) than that in the group with mixed infection having a median of 0.316 COI (interquartile range, 0.255 to 0.462) (Table 6). However, no significant difference was found for CMV IgG level, urine DNA load, blood DNA load, ALT, AST, and LDH.

**Table 6:** Clinical indicators between mixed *vs*. single type.

	Normal range	mixed infection	single virus strain	p
Number	\	12	51	\
CMV IgM	0.00–1.00COI	0.316(0.255,0.462)	1.585(0.404,7.348)	**0.003**
CMV-IgG	0.00–1.00U/mL	118.8(37.71,134.13)	128.10(36.61,2.45×10^3^)	0.241
Urine-DNA	0-500copies/ml	3.1×10^4^ (4.55×10^3^,1.3×10^5^)	2.1×10^4^ (250,4.63×10^5^)	0.971
blood-DNA	0-500copies/ml	<500	<500	0.457
ALT	7-40U/L	71.0(51.5,178.0)	75.0(52.8,129.0)	1.000
AST	13-35U/L	97.0(47.0,218.5)	81.0(50.0,138.5)	0.600
LDH	109-245U/L	432.0(349.8,596.0)	384.0(317.0,490.0)	0.404

The P value was obtained by using non-parametric Mann-Whitney U test. P < 0.05 indicated differences in clinical indicators between the mixed infection group and single virus strain infection group. The clinical indicators of the mixed infection group and single virus strain infection group did not present a normal distribution and the results were expressed by 50% quantile (25% quantile, 75% quantile).

### Distribution of UL144 and UL146 genotypes in different geographic regions

The distribution of cytomegalovirus genotypes differs with geographic region, as demonstrated by Fu *et al*. [36], Chen *et al*. [7], and Pignatelli *et al*. [37]. Thus, we also compared the distribution of UL144 and UL146 genotypes in our cohort with those previously reported, to determine whether geographic differences also exist. We found that the distribution of UL144 genotypes in our cohort (Table 7) was significantly different from the distribution in Illinois, USA [21], Europe [23, 26, 27, 38], Taiwan [7], and even Shenyang, China [5], but similar to the distribution in Tennessee, USA [19] and The Netherlands [39]. We noted, however, that the distribution of UL144 genotypes was similar in Japan and Taiwan [7, 22]. Similarly, the distribution of UL146 genotypes in our cohort was significantly different from the distribution in the UK [29], Poland [6], and Japan [40], in which G2 or G1 is predominant along with G7. However, the distribution in our cohort was similar to the distribution in the US [19] and Europe [41], in which G9 or G12 predominates along with G13 (Table 8).

**Table 7:** Distribution of HCMV UL144 genotypes in isolates from different cohorts and geographic regions.

Sample source	Genotypic distribution							
	A	B	C	AB	AC	1b	Total	P(versus hepatic involvement)
Hepatic Involvement (this study)	17	25	7				49	NA
Clinical isolates (Taiwan)(2015 Chen) [7]	55	34	27				116	**0.028**
1999 Lurain (USA) [21]	10	23	4	4		4	45	**0.028**
2006 Arav-Boger (Italy) [26]	12	14	7	1	3		37	0.163
2007 Mao (China) [5]	29	10	17			2	58	**0.001**
2008 Heo (USA) [19]	4	5	3				12	0.624
2010 Waters (Ireland) [27]	2	2	5	1	1		11	**0.003**
2014 Nijman (The Netherlands) [39]	4	5	3			1	13	0.244
2015 Oka (Japan) [22]	10	4	3				17	0.131
2015 Chen (CRC samples, Taiwan) [7]	22	13	8				43	0.127
2015 Brañas (Spain) [38]	9	16	14		9		48	**0.001**
2015 Paradowska (Poland) [23]	17	21	1	3			42	0.061

The P value was obtained by using the R × C chi-square test.

**Table 8:** Genotype distribution of HCMV UL146 in isolates from different cohorts and geographic regions.

Sample source	Genotypic distribution																P
	G1	G2	G3	G4	G5	G6	G7	G8	G9	G10	G11	G12	G13	G14	G15	Total	
Hepatic Involvement (this study)	3	1				1	3	2	15		2	7	7	1		42	NA
2008 Heo (USA) [19]	3						1	1	2		2	1	6	1	1	18	0.234
2005 Stanton (UK) [29]		11		1	1		9	8	7		2	6	3			48	**0.001**
2014 Paradowska (Poland) [6]	10	1			6		7	1	3			3	1			32	**0.000**
2008 Bradley (European) [41]	34	25	10	8	16	2	57	22	49	12	19	43	47	6		350	0.073
2010 Aguayo (Japan) [40]	14	2	4	3	4	3	1	4								35	**0.000**

The P value was obtained by using the R × C chi-square test.

### Correlation between UL144 and UL146 genotypes and clinical indicators

As shown in Table 9, there are differences in the distribution of the clinical indicators, AST (P = 0.028) and LDH (P = 0.046), among various genotypes of UL144. Further statistical analysis revealed that the concentration of AST and LDH in the UL144 genotype B group was obviously higher than that in the other two groups. However, UL144 genotype did not significantly correlate with viral load in the urine or blood, although median viral load was significantly higher in the urine than in the blood. In addition, UL144 genotype did not significantly correlate with serum CMV IgG and IgM, although CMV IgG was generally higher than normal (0.00−1.00 U/mL) in neonates infected with UL144 genotype A (77.435 U/mL, interquartile range 18.275 to 252.900 U/mL), genotype B (76.550 U/mL, interquartile range 37.670 to 144.000 U/mL), and genotype C (48.470 U/mL, interquartile range 16.306 to 221.150 U/mL). In contrast, CMV specific IgM was lower than normal (0.00−1.00 COI) in babies infected with genotype A (0.662 COI, interquartile range 0.369 to 2.123 COI) and genotype C (0.488 COI, interquartile range 0.261 to 2.415 COI), but higher than normal in babies infected with genotype B (1.180 COI, interquartile range 0.389 to 2.710 COI). The median for indicators of liver function, including total, direct, and indirect bilirubin, were all lower than normal in infected infants.

**Table 9:** Correlation of HCMV UL144 genotypes with clinical indicators of hepatic involvement in neonates.

	Normal range	A	B	C	P^a^
Number	\	17(34.69%)	25(51.02%)	7(14.29%)	\
Male^b^	\	10(58.82%)	18(72%)	3(42.9%)	\
CMV IgM	0.00–1.00COI	0.662(0.369,2.123)	1.180(0.389,2.710)	0.488(0.261,2.415)	0.569
CMV IgG	0.00–1.00U/mL	77.435(18.275,252.900)	76.550(37.670,144.000)	48.470(16.306,221.150)	0.757
Urine-DNA	0-500copies/ml	1.01×10^5^(1.62×10^4^,3.88×10^5^)	1.65×10^5^(1.93×10^4^,9.72×10^5^)	5.90×10^5^(2.5×10^2^,7.70×10^5^)	0.925
blood-DNA	0-500copies/ml	2.50×10^2^(2.50×10^2^,8.31×10^3^)	2.50×10^2^(1.25×10^2^,4.45×10^2^)	2.50×10^2^(2.50×10^2^,2.50×10^2^)	0.326
ALT	9-50U/L	72.5(65.50,97.00)	92.00(59.00,191.00)	49.00(19.00,100.00)	0.088
AST	15-40U/L	86.00(72.00,105.00)	101.00(53.50,175.00)	46.00(35.00,74.00)	**0.028**
LDH	109–245 U/L	335.50(288.25,442.75)	421.00(328.00,558.00)	309.00(0,420.50)	**0.046**
TBIL	6.8–34.2umol/L	4.90(2.925,9.525)	5.700(3.300,11.950)	5.800(5.300,7.600)	0.723
DBIL	1.7–8.6umol/L	1.650(0.750,2.900)	1.600(1.400,3.000)	1.400(0.900,3.150)	0.574
IBIL	4.8–25.0umol/L	3.900(1.650,6.400)	4.000(1.900,7.800)	4.400(3.750,5.100)	0.768
WBC	5–12*10^9^/L	11.80(8.78,15.65)	12.40(9.05,16.40)	11.00(7.84,16.40)	0.730
NE	0.5–0.7	0.29(0.18,2.90)	0.36(0.22,1.84)	0.26(0,5.96)	0.741
LY	0.2–0.4	0.75(0.66,4.41)	0.70(0.55,3.80)	7.10(1.90,10.21)	0.069
Hb	110-160g/L	118.00(106.00,123.25)	113.00(99.00,120.00)	120.00(102.50,127.00)	0.402
PLT	100–300*10^9^/L	335.00(250.75,400.50)	380.00(247.00,503.00)	262.00(106.00,477.50)	0.335
CRP	0-8mg/L	4.25(2.78,10.75)	4.80(1.90,9.85)	15.00(7.20,19.90)	0.352

ALT:alanine aminotransferase; AST: aspartate aminotransferase; LDH: lactate dehydrogenase; TBIL: total bilirubin; DBIL: direct bilirubin; IBIL: indirect bilirubin; WBC: white blood cell; NE: neutrophils; LY: lymphocyte; Hb: hemoglobin; PLT: platelet; CRP: c reactive protein. The nonparametric Kruskal-Wallis test was performed to compare clinical indicators in neonates infected with various genotypes. For clinical indicators showing distribution differences, we further adopted Nemenyi test to compare the differences between each other.

As expected, there are differences in the distribution of the clinical indicators, CMV IgM (P = 0.026), CMV IgG (P = 0.034), ALT (P = 0.019), and AST (P = 0.032, Table 10), among various genotypes of UL146. Further statistical analysis revealed that levels of ALT and AST suggested that G1 and G13 were associated with severe hepatic involvement. High viruria, lower viremia, and lower than normal total, direct, and indirect bilirubin were noted for UL146 genotypes, as observed for UL144. High rates of hepatobiliary disorder, as indicated by elevated hepatic transaminase and conjugated hyperbilirubinemia, were not observed, in contrast to results from other studies [42, 43].

**Table 10:** Correlation of the HCMV UL146 genotypes with clinical indicators of hepatic involvement in neonates.

	Normal range	G1	G7	G9	G12	G13	P^a^
Number	\	3(7.14%)	3(7.14%)	15(35.71%)	7(16.67%)	7(16.67%)	\
Male^b^	\	1	3	11	3	5	\
CMV IgM	0.00–1.00COI	4.74(4.36,10.85)	3.68(1.66,5.70)	0.68(0.31,2.68)	0.45(0.35,1.16)	2.50(1.04,8.68)	**0.026**
CMV IgG	0.00–1.00U/mL	17.58(16.22,25.01)	486.15(472.30,500.00)	68.73(49.91,116.70)	128.00(31.04,466.80)	50.85(28.46,149.90)	**0.034**
Urine-DNA	0-500copies/ml	1.00×10^4^(5.70×10^3^,4.61×10^6^)	1.80×10^5^(9.01×10^4^,6.40×10^5^)	3.00×10^5^(5.50×10^4^,9.50×10^5^)	3.80×10^5^(2.20×10^4^,1.30×10^6^)	9.65×10^4^(3.04×10^4^,5.60×10^5^)	0.903
blood-DNA	0-500copies/ml	2.50×10^2^(2.50×10^2^,9.13×10^3^)	<500	2.50×10^2^(2.50×10^2^,6.44×10^3^)	2.50×10^2^(2.50×10^2^,2.50×10^2^)	2.50×10^2^(2.50×10^2^,1.60×10^3^)	0.841
ALT	7-40U/L	236.00(156.00,314.00)	13.00(9.50,16.00)	74.00(61.00,101.00)	65.00(49.00,87.00)	159.00(46.00,194.00)	**0.019**
AST	13-35U/L	242.00(158.50,296.00)	30.00(22.50,32.50)	80.00(46.00,93.00)	83.00(52.00,101.00)	130.00(57.00,147.00)	**0.032**
LDH	109–245 U/L	646.00(384.00,908.00)	292.50(280.00,305.00)	395.00(312.50,446.50)	450.00(343.00,676.50)	347.00(319.75,600.50)	0.240
TBIL	6.8–34.2umol/L	7.100(4.900,7.800)	6.800(6.300,7.500)	4.750(3.225,7.525)	6.500(3.900,36.700)	7.800(3.300,15.600)	0.578
DBIL	1.7–8.6 umol/L	1.700(1.200,3.200)	1.800(1.600,3.050)	1.500(1.100,1.700)	2.000(1.550,19.550)	2.100(1.300,6.400)	0.305
IBIL	4.8–25.0 umol/L	3.800(2.900,4.600)	4.400(4.150,4.700)	3.200(1.800,5.375)	4.500(2.350,17.150)	5.700(2.500,11.500)	0.716
WBC	5–12*10^9^/L	14.00(12.10,16.40)	7.57(6.64,9.29)	10.15(7.98,14.83)	14.90(7.73,19.78)	9.90(8.60,15.20)	0.362
NE	0.5–0.7	0.36(0.27,1.15)	1.55(0.90,3.75)	0.26(0.18,0.77)	0.36(0.28,2.59)	2.22(0.25,4.44)	0.505
LY	0.2–0.4	0.75(0.63,4.09)	3.75(3.41,5.42)	0.71(0.61,1.42)	0.69(0.56,6.13)	5.85(0.66,7.25)	0.600
Hb	110-160g/L	118.00(114.50,118.50)	95.00(94.50,107.50)	114.50(102.50,121.00)	111.00(108.00,132.75)	113.00(96.00,122.00)	0.755
PLT	100–300*10^9^/L	368.00(302.00,492.50)	36.00(33.50,149.00)	307.00(255.75,428.00)	488.00(251.25,684.75)	380.00(312.00,389.00)	0.118

^a^ The nonparametric Kruskal-Wallis test was performed to compare clinical indicators in neonates infected with various genotypes. For clinical indicators showing distribution differences, we further adopted Nemenyi test to compare the differences between each other.

^b^ Abbreviations are the same as those in Table 9.

* represents mathematical operators “X”.

## Discussion

The UL144 transmembrane and cytoplasmic domains are conserved among clinical cytomegalovirus strains, although the ectodomain and signal peptide are highly variable [21, 23]. Accordingly, we noted that UL144 polymorphisms are concentrated in the 5' end of the gene, especially in cysteine-rich domain 1, which may bind the B- and T-lymphocyte attenuator protein. The activity of this domain may vary due to polymorphisms, resulting in a range of clinical symptoms and prognosis [13]. Notably, Heo *et al*. [19] analyzed cytomegalovirus from asymptomatic infected children and showed that UL144 did not mutate over two years even under pressure from host immunity, suggesting that UL144 diversity is not due to immune selection. In addition, several UL144 motifs such as N-glycosylation sites, protein kinase C phosphorylation sites, and cysteine-rich regions were conserved, suggesting that these domains are critical for infectivity [21]. Strong conservation of UL144 within the same genotype and its long−term stability in the host suggest that immune selective pressure helps maintain UL144 genotype.

The molecular epidemiology of American [19], Japanese [22, 40], Italian [6, 26], and Polish [23] isolates has been investigated based on UL144 and UL146. In addition, Waters *et al*. [27] suggested that congenital infection with UL144 genotypes A and C is serious and may lead to long-term clinical symptoms. Indeed, genotypes A and C generate markedly higher plasma viral loads than genotype B [27]. Similarly, Arav-Boger *et al*. [26, 30] demonstrated that genotypes A and C are associated with congenital cytomegalovirus symptoms, with genotype C identified in symptomatic patients only. Further, Pati *et al*. [44] found that genotype C was significantly more prevalent in symptomatic (6/20) than in asymptomatic infants (2/27). However, UL144 polymorphisms were also found to be unrelated to clinical symptoms in other studies [45, 46].

On the other hand, genotype B is usually detected in asymptomatic neonates [27]. Indeed, Paradowska *et al*. [47] showed that only genotype B was observed in asymptomatic children, while genotypes A and A/B were observed in symptomatic children, although the genotype did not significantly correlate with viral load in the blood and urine [39]. In contrast, genotype B was the most prevalent in our patients, who were symptomatic and were from a geographically distinct Chinese cohort, followed closely by genotype A. Genotype C was the least prevalent, and no recombinant strains were identified, as observed in previous surveys [27]. We noted that the prevalence of UL144 genotypes in our cohort was more similar to those reported by Heo *et al*. [19] for the US, and by Nijman *et al*. [39] for The Netherlands, than to those reported by Lurain *et al*. [21] for the US, by Mao *et al*. [5] for China, by Waters *et al*. [27] for Ireland, and by Branas *et al*. [38] for Spain. Collectively, the data indicate that the relative prevalence of UL144 genotypes was comparable between our cohort and the State of Tennessee, USA. In contrast, our results showed that newborns infected with genotype B had significantly higher AST and LDH than those infected with genotypes A and C, suggesting that genotype B is associated with severe hepatic involvement. We noted that Boppana *et al*. [48] and Yamamoto *et al*. [49] suggested that congenital infections are typically due to recurrent infection among pregnant women, either because of virus reactivation and/or further infection with other strains. Moreover, we found that genotype B only slightly stimulated CMV IgM (1.180, 0.389, and 2.710), but strongly stimulated CMV IgG, suggesting that genotype B infection is both short- and long-lived, while infection with genotypes A and C was generally long-lived. However, UL144 genotype did not significantly correlate with viral load in the urine or blood. Indeed, median viral load was <500 copies/mL in the blood for all genotypes, while median viral load in the urine was the highest at 5.90 × 10^5^ copies/mL for genotype C, and the lowest at 1.01 × 10^5^ copies/mL for genotype A.

In contrast, UL146 polymorphisms, mostly missense mutations, were found throughout the entire coding region. Significant differences in post-translational modification sites in the protein were also noted among dominant genotypes as well as in the predicted isoelectric point and molecular weight, which was also in contrast to UL144. These data indicate that UL146 may be more sensitive to host immune pressure. Further, the ELRCXC motif was present in 36 strains from G6, G7, G9, and G11−14, implying that this functional motif is essential. Indeed, He *et al*. [31] found this motif in most clinical strains obtained from congenitally infected patients with jaundice, megacolon, and microcephaly.

The relationship between UL146 genotypes and congenital infection has also been investigated [8, 19, 28]. For instance, Paradowska *et al*. [6] found that genotypes G1, G5, and G7 were prevalent in central Poland. In other studies in Europe, G1, G2, G7, G9, G12, and G13 were prevalent [41]. As a similar genotype distribution was observed in our cohort, we deem that G9, G12, and G13 are also prevalent in China, although He *et al*. [14] suggested that G1 and G2 are prevalent among Chinese infants. This discrepancy is probably due to regional differences and differences in the virulence of cytomegalovirus strains [6], as highlighted by Heo *et al*. [19], who found that G8, G11, and G13 were prevalent in the US. Notably, Paradowska *et al*.[6] found that, in 121 children with symptomatic infection, G7 and G5 were prevalent in postnatal infection, but G1 was predominant in congenital infection. Heo *et al*. [19] also found that G8, G10, and G12 are asymptomatic, although UL146 genotype did not correlate with symptomatic infection. Similarly, Arav-Boger *et al*. [28], Dolan *et al*. [8], and Hassan-Walker *et al*. [15] showed that no specific UL146 genotype was associated with clinical manifestation, but probably because of small sample size.

Median CMV specific IgM was normal in patients infected with genotype G9 and G12, but much higher than normal in patients infected with G1, G7, and G13. In addition, CMV specific IgG levels due to G7, G9, G12, and G13 were also elevated and comparable, suggesting that G9 and G12 infections were mainly long-lived, while G1, G7, and G13 were both short- and long-lived. Viremia was <500 copies/mL in patients infected with dominant genotypes, but the urine viral load was elevated and comparable among babies with G9, G12, and G13 infections. Levels of ALT and AST suggested that G1 and G13 were associated with severe hepatic involvement.

Mixed infection with multiple genotypes was detected in 15.19% of infants. Paradowska *et al*. [6] reported that mixed infection was approximately 7% in infants with postnatal infection and 11% in adults, but was not detected in neonates. Furthermore, Ross *et al*. [50] observed multiple genotypes in 39% (5/13) of urine, blood, and saliva samples. In another survey, mixed infection was also found in 59 infants (45%), but was not associated with symptoms [44]. Similarly, we did not observe a relationship between mixed infection and clinical outcome.

We found associations between specific genotypes and severe hepatic involvement based on comparisons among infants with congenital CMV infection and hepatic involvement. To make a conclusion, it would be important to investigate infants that have congenital infection with or without hepatic involvement in the same populations, otherwise the argument for its functional effects are still somewhat speculative. Yes, we previously reported on the polymorphisms of UL144 of congenitally infected asymptomatic neonates, but not analyze the polymorphisms of UL146 of congenitally infected asymptomatic neonates. This will be the subject of a future study. Moreover, It is important to define the significant associations of UL144 and UL146 genotypes and polymorphisms with congenital CMV with hepatic involvement or without hepatic involvement by increasing the numbers, and we will collect more cases for our future study.

In summary, we investigated the genotype distribution and polymorphisms in UL144 and UL146, which encode essential cytomegalovirus proteins, to assess whether polymorphisms are associated with hepatic involvement in infected infants. Because several UL144 and UL146 genotypes were detected, we speculate that various strains are transmissible from mother to child, with UL144 genotype A and B being the most prevalent along with UL146 genotypes G9, G12, and G13. We also confirmed that congenital infection with multiple strains occurs. In addition, UL144 genotype B and UL146 genotypes G1/G13 seemed to be associated with severe hepatic involvement. Taken together, the data show that different viral factors determine cytomegalovirus pathology in children [6]. However, the relationship between clinical indicators and UL144 and UL146 genotype needs further study. We emphasize that we are the first to report the prevalence of cytomegalovirus hepatic involvement in Chinese neonates.

## Supporting information

S1 Fig (A, B) Sequencing confirmed the specificity of PCR against human cytomegalovirus UL144 (A) and UL146 (B) in urinary sediment. The peak figures represented the full length sequences of UL144 and UL146, respectively, and the sizes of the bands were 531 bp and 357 bp, respectively.
(TIF)

S2 Fig Predicted isoelectric point (IP) and molecular weight (MW) of UL144 and UL146 protein. (A, C) Predicted molecular weights for UL144 (A) and UL146 (C). (B, D) Predicted isoelectric points for UL144 (B) and UL146 (D). The predicted objects of UL144 gene IP and MW were full length proteins. The predicted objects of UL146 gene IP and MW were mature proteins without the signal peptide sequence. Nonparametric Kruskal-Wallis test was used to compare isoelectric points and molecular weights of UL144 and UL146. P < 0.05 indicates statistical significance. * and ° represent the abnormal value deviated from the data set; the box plot in the figures from top to bottom represents the top edge value, 3/4 quantile, median, 1/4 quantile, lower edge value, respectively.
(TIF)

S1 Table Distribution of individual genotypes. (XLS)

S2 Table Homology of UL144 and UL146 nucleotide and amino acid sequences. (XLS)
