# Supplementary figures and images for: Polymorphisms and features of cytomegalovirus UL144 and UL146 in congenitally infected neonates with hepatic involvement
Source: PLoS One. 2017 Feb 21;12(2):e0171959. doi: 10.1371/journal.pone.0171959 (PMC5319779; doi:10.1371/journal.pone.0171959)

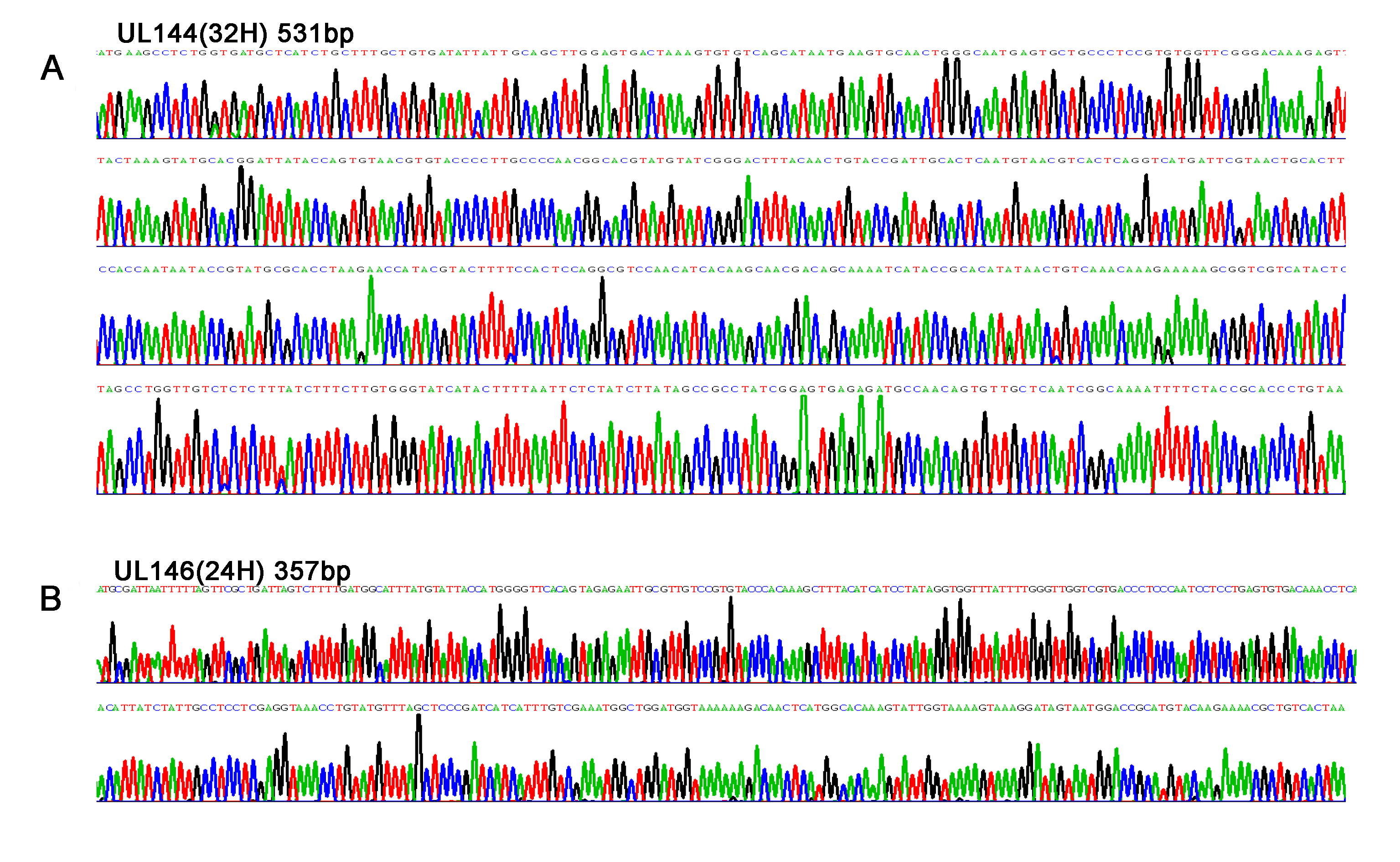

Supplement: S1 Fig — The peak figures represented the full length sequences of UL144 and UL146, respectively, and the sizes of the bands were 531 bp and 357 bp, respectively. (TIF) [file pone.0171959.s001.tif]
